# Climate change and climate change velocity analysis across Germany

**DOI:** 10.1038/s41598-019-38720-6

**Published:** 2019-02-18

**Authors:** A. Kosanic, I. Kavcic, M. van Kleunen, S. Harrison

**Affiliations:** 1 Ecology, Department of Biology, University of Konstanz, Universitätsstrasse 10, 78457 Konstanz, Germany; 2 Met Office, Fitz Roy Road, Exeter, EX1 3PB UK; 3 University of Exeter, Centre for Geography Environment and Society, Penryn, TR10 9FE UK

## Abstract

Although there are great concerns to what extent current and future climate change impacts biodiversity across different spatial and temporal scales, we still lack a clear information on different climate change metrics across fine spatial scales. Here we present an analysis of climate change and climate change velocity at a local scale (1 × 1 km) across Germany. We focus on seasonal climate variability and velocity and investigate changes in three time periods (1901–2015, 1901–1950 and 1951–2015) using a novel statistical approach. Our results on climate variability showed the highest trends for the 1951–2015 time period. The strongest (positive/negative) and spatially the most dispersed trends were found for Summer maximum temperature and Summer minimum temperatures. For precipitation the strongest positive trends were most pronounced in the summer (1951–2015) and winter (1901–2015). Results for climate change velocity showed that almost 90% of temperature velocities were in the range of 0.5 to 3 km/year, whereas all climate velocities for precipitation were within the range of −3.5 to 4.5 km/year. The key results amplify the need for more local and regional scale studies to better understand species individualistic responses to recent climate change and allow for more accurate future projections and conservation strategies.

## Introduction

The impact of current and future climate change on biodiversity and ecosystem services^1–3^ is reflected in changes in species distribution (including latitudinal, longitudinal and altitudinal changes), phenology, invasions, changes in populations, loss of genetic diversity and extinction^4,5^. The threats to biodiversity produced by climate change are magnified by increased fragmentation of natural habitats and regional introduction of new species and diseases^6,7^. In order to have a clearer perspective on biodiversity losses and extinctions as we have moved into the Anthropocene^8^ we need to understand the multiple components of climate that drive biodiversity loss, including past changes in regional and local temperatures, precipitation and extreme events^9^. Here, a regional focus is crucial as species-range reduction, population loss, species invasions and extinctions are spatially heterogeneous^10,11^, and these changes will affect regional and local ecosystem services and human wellbeing.

The lack of small spatial scale studies (i.e. local and regional) focussing on the magnitude, direction and velocity of climate change makes it difficult to obtain a clear picture of how different species will respond to future climate conditions. Such research is missing in many parts of the world due to a lack of long-term (more than 50 years) historical weather records and the difficulties of trend detection as climate data are often compromised by changes in observation techniques and changes in instruments or station relocations^12^. Without data homogenization^13^ historical and archive records can show trends unrelated to climate variability which obscure our understanding of biological responses to recent climate change. In addition, magnitude and direction of temperature change are much easier to detect than precipitation change due to high spatial and temporal variability^14^.

Despite these issues, the question of the attribution of biodiversity responses to climate change is one of the key unanswered questions in ecology^15,16^. Although attribution in climate-change research has made considerable progress, and although we are able to attribute certain specific weather events to anthropogenic climate change^17^, we are still not able to attribute individualistic species responses (both terrestrial and aquatic species) to climate variability, particularly at regional or local scales^16^. Addressing this ‘trend attribution’ question in ecological research is of crucial importance. What we mean by ‘trend attribution’ here is not aiming to attribute biological changes to greenhouse gasses or natural climate variability but to better understand biotic responses to recent climate change regardless what the cause at the local or regional scale might be. Achieving this would allow a more complete understanding of species sensitivity to climate change and provides the basis for the conservation of regionally and locally important species, such as key species or those crucial for ecosystem functioning and services^18^. It would also help to better understand the ecology and patterns of species invasions on local and regional scales and to identify if alien species have profited from recent climate change^19,20^. Here we use small scale climate trend analysis from Germany to provide a stepping stone in order to better understand abiotic responses to climate trends across small-spatial scales.

Beside trend analysis to estimate the magnitude and direction of climate change, the most commonly used metric to estimate the potential impact of climate change on biodiversity is climate change velocity^21^. Climate velocity has been defined as the speed at which species will need to migrate in order to stay in the same enveloped climatic condition^22^. Previous studies analysing climate change velocity have aimed to better understand species extinction risk^23^ or the expected vector of past and future biological migrants^24,25^. It is known that some plant species only partly track trends in recent climate change^25^, and the velocity of how fast they should move has differed from the observed migration. Such ‘migration lag’ in plants can be related to changes in multiple climate variables (i.e. temperature, precipitation) and different types of lags (i.e. dispersal lag, establishment lag and extinction lag)^26,27^. This could impact various trophic levels, for example change animal migration rates caused by the loss of nutrition or habitat^27^. An example, by Chivers *et al*.^28^, has shown that phytoplankton responses to climate change were highly variable; some taxa closely tracked climate velocity, while other taxa showed no response. Such research is starting to explain responses to climate change at the species level, which is necessary to enable us to make realistic projections of species change in the current and the next century.

In order to better recognise individual species’ responses to climate forcing, we require studies on past and present regional and local climate variability and velocity, as well as the studies that combine both climate analysis and studies on species range changes, migration distances, direction and velocity. Although, the latter is not an objective of this study, analysing multiple aspects of climate change and climate velocity is necessary to highlight species’ climate sensitivity (across different taxonomic groups), their migration capacity and to distinguish the impact of climate from other environmental drivers^29^. Such studies will also help to more accurately identify species resilience to climate change associated with genetic variation, phenotypic plasticity, dispersal ability or interactions with other taxa.

As suggested by recent studies^30^, extensive regional and local climate change research is missing in many regions yet, and this is probably the only way to fully understand the response of ecological communities or individual species to climate drivers, including the identification of potential climate refugia^31,32^. Here we present the most detailed assessment that combines the analyses of seasonal climate change magnitude, direction and the recent velocity of climate change throughout the 20^th^ and 21^st^ century. In this study we introduce three novel adaptations of the method for climate velocity calculation of Burrows *et al*.^24^, as follows: (1) Temporal trends are calculated by a non-parametric Mann-Kendall analysis instead of linear regression; (2) Spatial gradients in long-term median instead of average quantities were used to be consistent with the approach to temporal trends; (3) A novel approach was used to constrain occurrences of near-zero values of spatial gradients. The benefit of these adaptations is an increased robustness in dealing with data distributions potentially skewed by extremes to which an average based approach is more sensitive. In this study we use Germany as an example, because it has excellent availability of long-term weather records, including one of the world’s longest precipitation records. Furthermore, we assess  that the response of species to seasonal climate variability and velocity could be more easily detected, rather than  to coarser metrics such as annual changes in temperature and precipitation, as changes in species distribution are rarely controlled by the mean annual temperatures^27^.

## Methods

We analysed the seasonal climate change direction, magnitude and velocity across the whole of Germany, an area of 357 168 km^2^. Germany has a cold temperate climate with regional variations in annual mean temperature, between 9–11 °C. Precipitation is also spatially and temporarily variable with the lowest annual precipitation in the northeast (450 mm) and the highest in the south (970 mm).

We used local scale (1 × 1 km) weather records for 1901–2016 from the Deutscher Wetterdienst (DWD), providing monthly mean maximum temperature at 2 m above the ground, minimum temperature at 2 m and total monthly precipitation. All datasets were quality checked and homogenized by Deutscher Wetterdienst and, therefore, tests for homogeneity of variance and homogeneity adjustment were omitted^33,34^. Yearly data were grouped into the seasons winter (December, January, February; DJF), spring (March, April, May; MAM), summer (June, July, August; JJA) and autumn (September, October, November; SON). The analyses were conducted for the period 1901–2015, and for the periods 1901–1950 and 1951–2015 separately, as the anthropogenic impact on Earth’s climatic system has been shown to be more pronounced since the 1950s^7,8,35^. As the winter season starts in December, the relevant ends of the periods were moved to February 1951 and February 2016, respectively. There are gaps in the data in the period between 1935 and 1946 for maximum and minimum temperatures, except for the minimum temperature in autumn. All grid points affected by the missing data were excluded from all the analyses.

As in Kosanic *et al*.^36^, we used a Mann-Kendall test in order to detect positive or negative trends in seasonal temperature and precipitation change. This test is considered to be a robust non-parametric method for the detection of monotonic trends^36–39^. By implementing the trend analysis for all complete time series, we generated maps showing areas with trends for all three variables (°C/year for temperature and mm/year for precipitation) at a significance level p ≤ 0.05 for all periods mentioned above (control (1901–1950), reference (1951–2015), and whole (1901–2015)). Sen’s slope estimator, a non-parametric version of linear regression based on the median rate of change among all data points, was used to show the direction and magnitude of climate trends^40^ (see Supplementary Information for more details).

The velocities of climate change (km/year for all variables) were calculated for 1951–2015 following the methods of Loarie *et al*.^21^ and Burrows *et al*.^24^ as ratios of Sen’s slope estimators for temporal trends and absolute values of spatial gradients (°C/km for temperature and mm/km for precipitation), with the direction of the velocity oriented along the direction of spatial gradient. To be consistent with our approach to temporal trends, we calculated median instead of the mean long-term spatial gradients (see Supplementary Information for more details). Climate change velocities calculated by this method are often named “local” as they consider spatial variability within the immediate neighbourhood of a location^24^ and, when combined with a fine spatial resolution of data, they give fine grain estimates of residence times and velocities of climates^21^. On the other side, as pointed out in^21,22,24^, the values can approach infinity in areas of small spatial gradients, such as flat terrain, and underestimate values in mountainous terrain where spatial gradients can be several orders of magnitude larger (constraining near-zero values of spatial gradients is described in Supplementary Information^41–43^). Hence species in large topographically homogenous areas would have to move at high speed to keep pace with climate change, whereas in mountain regions the calculated migration rate might be lower than is actually required.

## Results

### Climate change analysis

**Figure 1:**
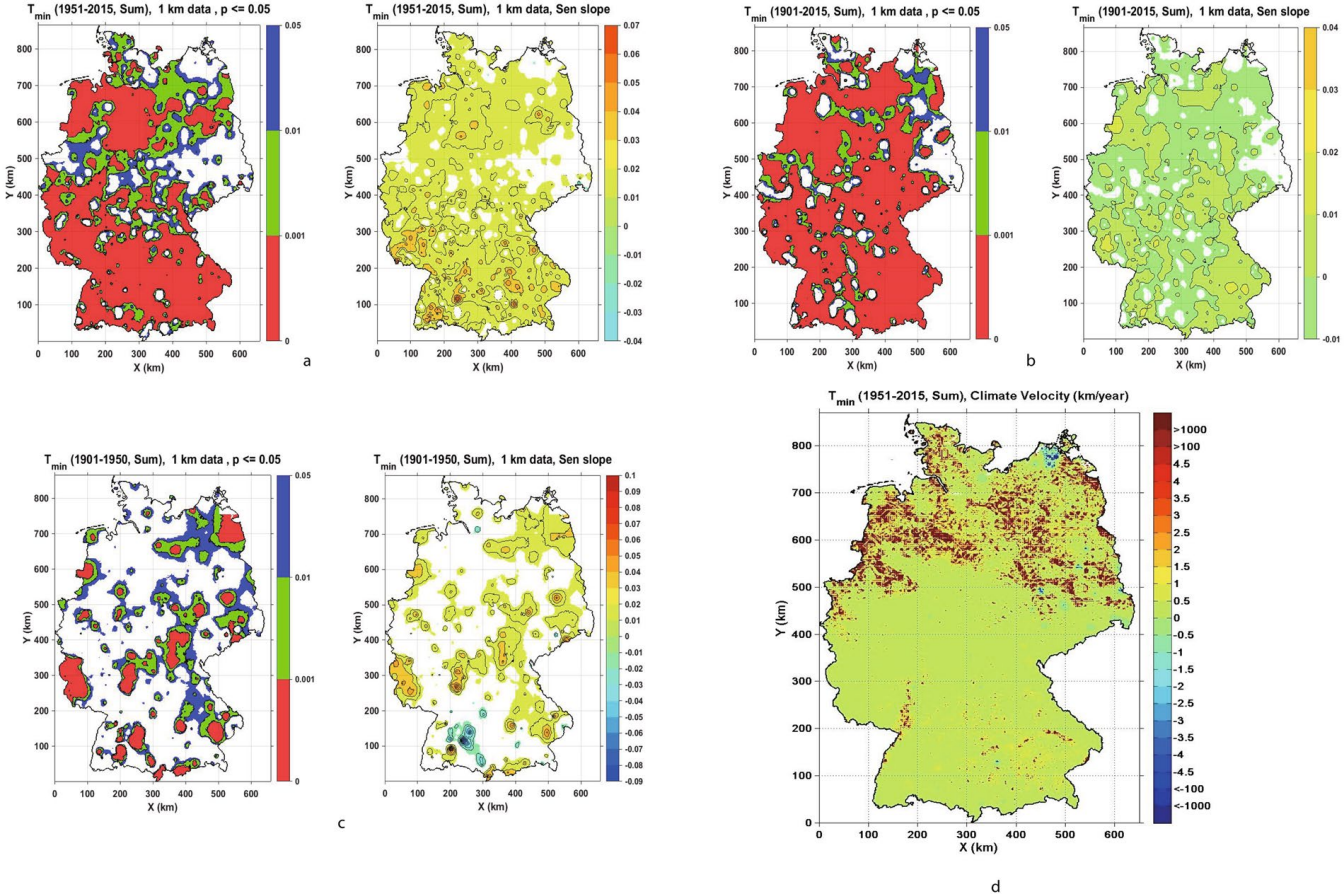
(**a**–**c**) Seasonal Trend analysis for Summer minimum temperature (Tmin) for the periods 1901–1950, 1901–2015 and 1951–2015, respectively, showing values of significance level p ≤ 0.05 (left panels: p ≤ 0.001 red, 0.001 < p ≤ 0.01 green and 0.01 < p ≤ 0.05 blue) and Sen’s slope (right panels). (**d**) Climate change velocity for Summer minimum temperature (Tmin) for period 1951–2015.

**Figure 2:**
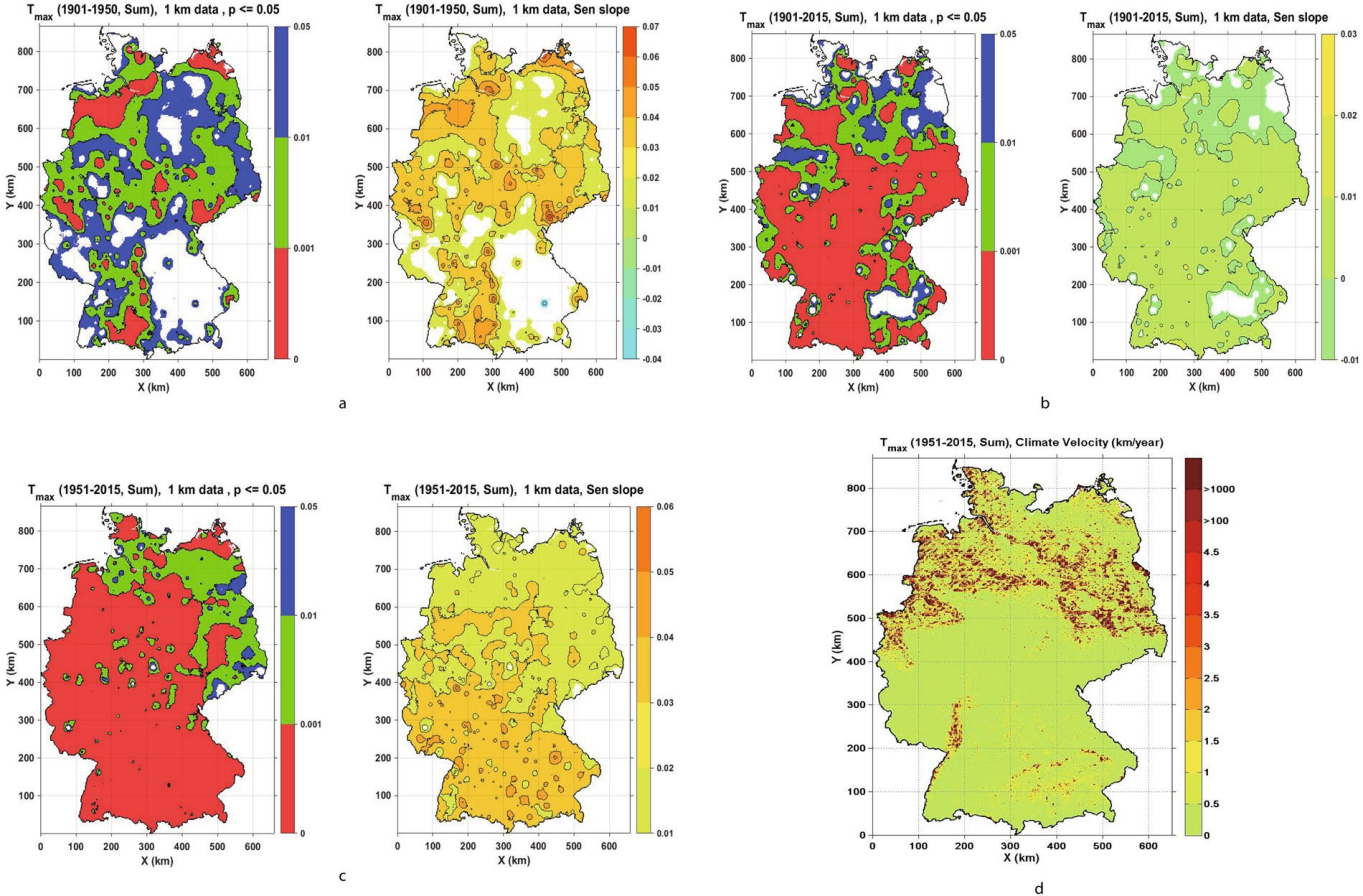
(**a**–**c**) Seasonal Trend analysis and climate change velocity for Summer maximum temperature (Tmax) for the periods 1901–1950, 1901–2015 and 1951–2015, respectively, showing values of significance level p ≤ 0.05 (left panels: p ≤ 0.001 red, 0.001 < p ≤ 0.01 green and 0.01 < p ≤ 0.05 blue) and Sen’s slope (right panels). (**d**) Climate change velocity for Summer maximum temperature (Tmax) for period 1951–2015.

**Figure 3:**
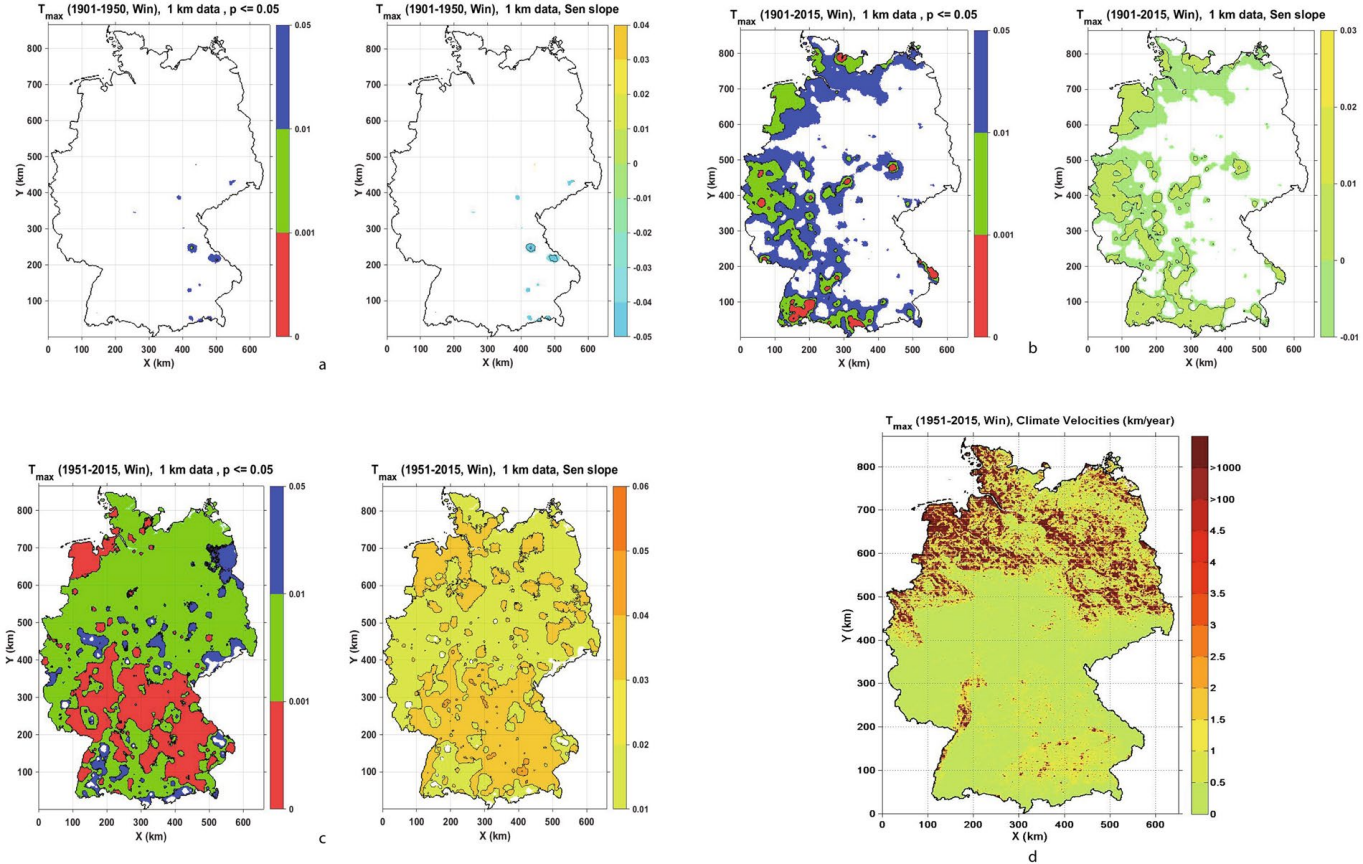
(**a**–**c**) Seasonal Trend analysis and climate change velocity for Winter maximum temperature (Tmax) for the periods 1901–1950, 1901–2015 and 1951–2015, respectively, showing values of significance level p ≤ 0.05 (left panels: p ≤ 0.001 red, 0.001 < p ≤ 0.01 green and 0.01 < p ≤ 0.05 blue) and Sen’s slope (right panels). (**d**) Climate change velocity for Winter maximum temperature (Tmax) for period 1951–2015.

Temperature trends were highest for the time period 1951–2016 throughout the seasons. The strongest (positive and negative) trends and the most spatially dispersed ones were for Summer maximum and Summer minimum temperatures (Figs 1 and 2a–c)). Significance levels of temporal trend strength are shown in the left panels (*p* ≤ 0.001 red, 0.001 < *p* ≤ 0.01 green and 0.01 < *p* ≤ 0.05 blue), and the magnitudes of statistically significant (*p* ≤ 0.05) Sen’s slopes are shown in the right panels. The results for Summer minimum temperature showed both positive and slightly negative trends in all three examined periods (i.e.1901–2015; 1901–1950; 1951–2015), whereas the spatial coverage across Germany was the largest for Summer minimum temperature in the period 1901–2015. For the winter season, the most significant positive trends were detected for the Winter maximum temperature in the period 1951–2015 for most of Germany (Fig. 3a–c)). The season that follows next in showing the highest spatial coverage (in number of analysed grid cells) is spring with the highest positive trends for maximum temperature in 1951–2015. For Spring minimum temperature, there is a relatively small number of grid points with positive trends, and they are mainly concentrated in North-West Germany. Autumn is the season with the least predominant trends in maximum and minimum temperature. For Autumn maximum temperature there were positive trends between 1901–2015 for grid points in South-West Germany. The trends in precipitation are considerably weaker for all observed seasons when compared to temperature results. There are small, nearly all positive, trends for Winter precipitation in the 1901–2015 period evenly distributed across Germany. Similar results for Autumn precipitation show more spatially distributed mainly positive with some negative trends for century long data covering North-West and South-East Germany. However, the trends for the period 1951–2015 showed higher positive magnitudes but lower spatial coverage. Summer and spring season showed the least change in precipitation across Germany, and mainly for the period 1901–2015. For further details of these results, see the Supplementary Information.

### Climate change velocity

To briefly recap, climate change velocities were calculated as ratios of Mann-Kendall temporal trends and absolute values of spatial gradients, so negative velocities correspond to a negative climatic trend and positive ones to a positive climatic trend. Since not many statistically significant temporal trends were detected in the period 1901–1950, we focused on estimating climate velocities for the recent period from 1951 to 2015. As described in the Supplementary Information, we categorised values of climate change velocities for each variable and season by analysing their respective histograms. Almost 90% of climate change velocities for both maximum and minimum temperature were contained within the range of (−0.5 km/year to 3 km/year) (Figs 1–3d). The majority of those values for maximum temperature are in the range −0.5 km/year to 3 km/year and for minimum temperature in the range (−0.5 km/year to 1.5 km/year), (Tables 1 and 2, Supplementary Information). Spring velocity for Tmax showed the highest values of 1.5 km/year, and this covers 3.58% of Germany, whereas for Tmin the highest positive velocity is detected for the Winter season, covering 16.88% of Germany. Climate change velocities for both minimum and maximum temperatures were higher in northern Germany and in topographically low elevation areas, whereas in mountain areas the velocities were lower.

All climate velocities for precipitation were contained within the range of (−3.5 km/year to 4.5 km/year). Furthermore, velocity showed negative values for summer and spring covering 74.73% and 36.83% of the analysed area Table 3, Supplementary Information. Majority of values for autumn and winter were positive (98.23% and 96.15% of the analysed area, respectively). The highest positive value of velocity for precipitation was detected for autumn (4 to 4.5 km/year) and after that spring (3 km/year to 3.5 km/year).

## Discussion

In our trend analysis of the local and regional scale of monthly temperature and precipitation, we analysed three periods (1901–1950; 1901–2015; 1951–2015) and detected positive and negative trends in climate variables for all four seasons. The spatial coverage and the magnitude of positive trends were highly uneven, but most pronounced in the latter (third) period (1951–2015). Our findings on climate change across Germany are thus consistent with previous research that shows an increase in seasonal temperatures across Europe since the mid-20^th^ century^44^. Negative trends on the other hand were less spatially pronounced and were restricted for the first part of the 20^th^ century. This is in contrast with findings of Matiu *et al*.^45^, who detected both warming and cooling trends for southern Germany (station Hohenpeissenberg). They detected decreasing trends for spring and autumn and increasing trends for winter and summer. This illustrates the high spatial and temporal variability of temperature and precipitation, and highlights the necessity for local scale studies.

The climate in Germany is influenced by atmospheric flows coming from the Atlantic, driven by changes in the North Atlantic Oscillation (NAO)^46^. NAO anomalies cause large shifts in regional winter temperatures and extreme weather conditions^47^. The strongest impact is visible through warmer than average winter temperatures during the positive mode of the NAO, and with an opposite effect during the negative mode of the NAO^48^. Here we found trends that are more spatially pronounced over the course of the whole 20^th^ century than for the first or the second half of the 20^st^ century. This might be due to the fact that long-term time series have a higher statistical power, but also to the fact that time series could be affected by extreme values and variances within the dataset^49^.

Winter and autumn precipitation trends were strongly dependent on the duration of the analysed time series and are more predominant and spatially heterogeneous for the 1901–2015 period than for 1901–1950. Although changes in precipitation are highly spatially and temporarily variable, and trends are less spatially homogenous, we found an increasing trend of Summer precipitation for northern Germany, which has been previously mainly detected for southern Europe^50^ and for east-central Germany^51,52^. Previous studies detected an increasing Summer precipitation for some parts of northern Europe^53,54^, which is only to some extent consistent to our findings, as we detected positive trends in some parts of central and south west Germany.

Our analysis calculated climate change velocity since the 1950s and estimates the rate of climate change that organisms will have to respond to^55^. The results show higher values of climate change velocity in Germany than previous studies that used coarser data. Different results may be due to the different datasets and higher data resolution we used and to our approach in calculating the main constituents of climate velocities. Using the Mann-Kendall trend analysis instead of linear regression and medians instead of means of the spatial gradients decreases the sensitivity to a potentially skewed distribution of data. The highest velocity rates across Germany were calculated for lowland areas and the lowest rates in the mountain regions. Similar results were found for California by Loarie *et al*.^21^. This is because the spatial gradient of temperature change is the highest on mountain slopes and no large movements up or down the slope are required, thus resulting in a large change in temperature but low velocity. The opposite is true for the lowland areas, where large movements are required across geographical areas requiring higher velocity^21,56,57^. Precipitation change velocity is also lowest in the mountain areas due to the orographic effect^21^. Although the limitation of this method is that it can underestimate constant climate conditions in the mountain areas, it has nevertheless been accepted as a baseline method and it could be complemented with other climate change velocity metrics (i.e. biotic velocity)^58^. Nevertheless, climate change velocity results could, to some extent, depend on climatic variables that are used, spatial resolution of the data and to the parameter that defines analogue climates^58^.

A commonly used method for limiting occurrences of near-zero spatial gradients in calculation of climate velocities is adding a small random noise to their values^21^. In this random noise method, the user directly determines the range of generated “uncertainties”, usually small in magnitude relative to the signal for which they are generated. Here we use a bootstrap method^42,43^ that, on the other hand, requires user input about the number of generated time series from which the uncertainty range is calculated and not the range itself. The assumption about optimal calculation can be tested by adding more generated series to the analysis, hence making bootstrap more mathematically rigorous (less arbitrary) than the random noise generation. In any case, the choice of method for limiting near-zero spatial gradients can significantly affect only the extreme values of climate velocities, usually related to lowland areas, and therefore we recommend the use of a bootstrap method in calculation of near-zero spatial gradients.

Data based studies like the one we present here can help to further explore the causal links and attribution of species shifts across multiple taxa to local and regional climate variability. Species from multiple taxonomic groups have different climatic requirements and heterogeneous responses to climate change (i.e. either on the individualistic or population level) that are not fully understood^16,59^. Some studies have used biotic velocity (metrics developed based on climatic niche models)^58,60^, and have analysed plant species vulnerability (on a population level) to local climate change velocity and their migration abilities (i.e. dispersal abilities)^61^. Still, such studies use species distribution data from range maps which are scale dependent, and this could overestimate the presence of a particular species. In other words, they do not use species occurrence data^58^ and, in order to better understand to what extent species have followed previous climate change velocity at the local and regional scales, we need studies based on occurrence data. Such studies can serve as a guide to estimating the rate of future ecological migrations driven by climate velocity.

## Conclusion

With the continued rapid climate change that is anticipated for coming decades, fine scales studies on a seasonal climate change and velocity analysis over the past century and in particular over the past 60–70 years are necessary to understand biological responses of different taxonomic groups and to identify the most vulnerable species, and possible climate refugia^62^. Such regional/local scale climate change baseline studies do not only open the door for more necessary collaboration between climate change scientists and ecologists, but they also allow better clarifications between recent biodiversity changes and their impact on ecosystem services^12,63^. This could lead towards more accurate future projections and to more effective policy and biodiversity management planning^12,57,64,65^. Therefore, we argue for more combined research on detecting the magnitude/velocity of seasonal climate variability (as many species do not respond to the mean annual temperature or precipitation change)^29^ versus observed shifts in the species’ geographical distribution at local and regional spatial scales.

## Supplementary information

Climate change and climate change velocity analysis across Germany
