# Supplementary material for: Climate change and climate change velocity analysis across Germany
Source: Sci Rep. 2019 Feb 18;9:2196. doi: 10.1038/s41598-019-38720-6 (PMC6379444; doi:10.1038/s41598-019-38720-6)
Supplement: Supplementary file 1 — Climate change and climate change velocity analysis across Germany [file 41598_2019_38720_MOESM1_ESM.docx]

**Climate change and climate change velocity analysis across Germany**

**A. Kosanic^1*^, I. Kavcic^2^, M. van Kleunen^1^, S. Harrison^3^**

^1*^Ecology, Department of Biology, University of Konstanz, Universitätsstrasse 10, 78457 Konstanz, Germany, *Correspondence to: [aleksandra.kosanic@uni-konstanz.de](mailto:aleksandra.kosanic@uni-konstanz.de)

^1^MvK. Department of Biology, University of Konstanz, Universitätsstrasse 10, 78457 Konstanz, Germany

^2^IK.Met Office, Fitz Roy Road, Exeter EX1 3PB, UK

^3^SH. University of Exeter, Centre for Geography Environment and Society,

Penryn, TR10 9FE, UK

**Supplementary Information**

**Study region and data**

For the analysis, we used the Deutscher Wetterdienst (DWD) local scale (1x1 km) weather records for 1901-2016 providing monthly mean maximum temperature at 2 m above the ground, minimum temperature at 2 m and total precipitation, which can be downloaded from <ftp://ftp-cdc.dwd.de/pub/CDC/grids_germany/monthly/>. The temperature data are stored in integer format in units of 1/10 °C and thus need to be converted (divided by 10) before further analysis (to get units of 1°C). The precipitation data are stored in integer format in units of mm. As mentioned in the paper, no homogenization was required.

Each monthly data file contains 654 x 866 = 566 364 grid points. The grid points outside the border of Germany were identified by having the missing data identifier -999 (replaced by NaN in MATLAB analysis) for the entire analysed period. The number of grid points within the borders of Germany is *N* = 358 303.

There are gaps in the data (also marked by -999 and replaced by NaNs in the MATLAB analysis) for maximum and minimum temperatures in the period between 1935 and 1946. Time series of maximum temperature are not complete with gaps in 1946 for 123 grid points in the Winter, Spring and Summer seasons, as well as 28 grid points in the Autumn season. Time series of minimum temperatures lack values for the year 1946 in Winter and Spring (14 grid points for both) and the years 1935, 1942-1946 in Summer (1125 grid points). All grid points affected by the missing data were excluded from all the analyses, as they were localised to a small square region in the North-East of Germany and accounted for only 0.004%-0.3% of the grid points. These gaps only affect the 1901-1950 period for maximum and minimum temperatures. Seasonal precipitation contains the full set of data, as well as the reference period from 1951 to 2015 for all three variables.

To summarise, all *N* time series were used for the analyses (minus gaps in the above described cases), with lengths of 150, 195 and 345 values per control, reference and whole period, respectively.

**Mann-Kendall trend analysis**

The Mann-Kendall trend analysis was conducted in MATLAB for each of the *N* time series within Germany’s borders, excluding the grid points with missing data as described above. J. Burkey’s [86] function ktaub.m was used to calculate the Mann-Kendall Tau with Sen's slope and respective confidence intervals. The value of the Sen’s slope describing strength and sign of a temporal trend is hereafter denoted with $T$ and its units are °C/year for temperature and mm/year for precipitation. For more general details on Mann-Kendall trend analysis and the calculation of the non-parametric Sen’s slope please see references ^1-4^. The computed variance in the function accounts for ties in the data and time indices, as well as for anomalies in trends (see [68] for more details and references). The seasonal data was grouped by the year, as ktaub.m can be sensitive to data not being spaced equally over time ^4^.

We analysed the residuals of datasets for each variable for serial correlation. The residuals of the datasets, calculated by detrending the seasonal time series using their Sen’s slope estimators, were analysed for serial correlation for each variable. We used Durbin-Watson and Ljung-Box tests at the significance level p ≤ 0.05 to be consistent with the significance level used in the Mann-Kendall analysis. There was some serial correlation present in the precipitation residuals however further analysis confirmed that those grid cells were excluded by the significance testing in the Mann-Kendall analysis.

**Climate-change velocity**

The velocity of climate change was calculated for 1951-2015 following the method of Burrows et al. ^5^, but with three main differences in approach when compared with the previous studies ^5-7^. The first one, as described above, was the calculation of the temporal trends by the Mann-Kendall method instead of linear regression, as it has been shown that non-monotonic trend detection methods are more appropriate for climatic data ^1-3^. We started with Burrows et al. ^5^ 9-points method to evaluate the long term North-South (NS) and West-East (WE) spatial gradients (°C/km for temperature and mm/km for precipitation) for seasonal data on a year-by-year basis (excluding any missing values along the Germany border). The method is illustrated in Figure S1. Each component of the spatial gradient in a focal point *e* is calculated using its 3x3 neighbourhood (Figure S1 a)). NS gradients were calculated as weighted averages of North-South differences (Fig. S1 b)) divided by distance between points (1 km) with weights of 2 for adjacent points distances (e.g. *e – b*) and 1 for diagonal points distances (e.g. *c - f*). WE gradients were calculated in a similar manner using weighted averages of West-East differences divided by distance (Fig. S1 c)).

*a b c*

*d e f*

*g h i*

*a - d b - e c - f*

*d - g e - h f - i*

*1 2 1*

*1 2 1*

*b - a c - b 1 1*

*e - d f - e 2 2*

*h - g i - h 1 1*

*a) b) c)*

*Figure S1: Illustration of Burrows et al. ^5^ 9-points method for calculating spatial gradients. a) Focal point e and its neighbourhood, b) NS differences and weights, c) WE differences and weights*.

As in ^5^, the vector sum of NS and WE gradients ($\vec{S}$, Eq. 1.1) was used to calculate the magnitude or absolute value of the spatial gradient ($S$, Eq. 1.2) and the angle between them ($\alpha$, Eq. 1.3) to calculate the direction of the gradient.

$\vec{S}=S_{WE}\vec{i}+ S_{NS}\vec{j}$ (1.1)

$S=\left| \vec{S} \right|=\sqrt{S_{WE}^{2}+ S_{NS}^{2}}$ (1.2)

$\alpha= \tan^{-1} \frac{S_{NS}}{S_{WE}}$ (1.3)

The second difference to ^5^, in order to be consistent with our approach to temporal trends, was using the medians of yearly absolute values of spatial gradients ($S$, Eq. 1.2) to calculate the velocities of climate change instead of the averages as in previous studies ^5-7^. Finally, the velocities of climate change ($V$, Eq. 2, km/year for all variables) for each grid point (excluding points with incomplete time series as explained above) were calculated as ratios of Sen’s slope estimators for temporal trends ($T$) and median values of $S$ for a given period (1951 – 2015 in this work):

$V= \frac{T}{S}$ (2)

As pointed out in [40, 42, 43], $V$ can approach infinity in areas of small spatial gradients. A usual approach to constraining occurrences of near-zero values of spatial gradients in the denominator is adding uniformly distributed random noise to all grid points (e.g. Loarie et al. ^7^). In this method the user directly determines the range of generated noise, usually small in magnitude relative to the signal for which the noise is generated, which seems somewhat arbitrary. Climate variables are usually determined up to some uncertainty, whether by measurement or by calculation, and are expressed in terms of [*x – ε_1_*, *x + ε_2_*]. Therefore, it seemed natural to limit the near-zero values of spatial gradients by the uncertainty in their time series for each grid point in the domain. As a third difference in the approach of calculating climate change velocity we used the well-known and tested bootstrap method to generate the uncertainties [43,47]. Furthermore, we tested and compared two bootstrap methods to estimate uncertainties and confidence intervals for median spatial gradients. One was the MATLAB built-in bootstrap method, which created new time series of spatial gradients by randomly sampling individual values in the original time series. The other was the bootstrap method with automatic block length selection^8-10^, which randomly sampled blocks of values in the original time series. Uncertainties were calculated as spread (standard deviation) of medians of time series of spatial gradients, and confidence intervals as percentiles accounting for 95% of the data (p ≤ 0.05 to match the approach in temporal trends). Both methods were employed on stationary time series of spatial gradients (calculated by detrending the original time series using their Sen’s slope estimates from Mann-Kendall trend analysis). We used the widely recommended number of 1000 generated time series for the analysis of each original time series in both methods, after verification on samples of data that the accuracy of the uncertainty estimates was not significantly improved by including more generated series in the analysis. The results for both methods were very similar, and therefore MATLAB bootstrap was used as it proved to be computationally more efficient.

After constraining the spatial gradients, we analysed histograms of calculated climate velocities for each season and variable to facilitate categorisation of their values (Tables 1, 2 and 3). About 90% of climate velocities for both maximum and minimum temperature, as well as all values for precipitation, were contained within the range of -3.5 km/year to 4.5 km/year. The majority of the 10% of absolute values of velocities for maximum and minimum temperatures that fell outside of the range (-3.5 km/year to 4.5 km/year) were larger than 1000 km/year, which indicates near-zero spatial gradients in the denominator (Tables 1 and 2). Hence, the resolution of climate velocity values in plots was set to 0.5 km/year for values in the range of -4 km/year to 4 km/year, whereas the rest were stored by absolute values > 100 km/year and > 1000 km/year.

| **T_max_** | **Winter** | | **Spring** | | **Summer** | | **Autumn** | |
| --- | --- | --- | --- | --- | --- | --- | --- | --- |
| **C (km/year)** | **N_C_** | **P (%)** | **N_C_** | **P (%)** | **N_C_** | **P (%)** | **N_C_** | **P (%)** |
| ( -∞, 1000) | 0 | 0.00 | 0 | 0.00 | 0 | 0.00 | 10 | 0.00 |
| [ -1, -0.5 ) | 0 | 0.00 | 0 | 0.00 | 0 | 0.00 | 1 | 0.00 |
| [ -0.5, 0 ) | 0 | 0.00 | 0 | 0.00 | 0 | 0.00 | 2534 | 0.71 |
| [ 0, 0.5 ) | 217768 | 60.78 | 232577 | 64.91 | 265944 | 74.22 | 305697 | 85.32 |
| [ 0.5, 1 ) | 67979 | 18.97 | 68337 | 19.07 | 48862 | 13.64 | 11974 | 3.34 |
| [ 1, 1.5 ) | 27131 | 7.57 | 19630 | 5.48 | 16578 | 4.63 | 134 | 0.04 |
| [ 1.5, 2 ) | 1478 | 0.41 | 12838 | 3.58 | 1770 | 0.49 | 2 | 0.00 |
| [ 2, 2.5 ) | 52 | 0.01 | 612 | 0.17 | 118 | 0.03 | 0 | 0.00 |
| [ 2.5, 3 ) | 0 | 0.00 | 175 | 0.05 | 1 | 0.00 | 0 | 0.00 |
| [ 3, 3.5 ) | 0 | 0.00 | 8 | 0.00 | 0 | 0.00 | 0 | 0.00 |
| [ 1000, ∞ ) | 43888 | 12.25 | 24119 | 6.73 | 25023 | 6.98 | 37944 | 10.59 |

***Table 1:*** *Histograms of values of climate velocities for maximum temperature (T_max_, all four seasons in the period 1951 – 2015). Here N_C_ and P denote the total number of grid points and the percentage of grid points containing values for each category within the border of Germany (excluding any missing values along the border).*

| **T_min_** | **Winter** | | **Spring** | | **Summer** | | **Autumn** | |
| --- | --- | --- | --- | --- | --- | --- | --- | --- |
| **C (km/year)** | **N_C_** | **P (%)** | **N_C_** | **P (%)** | **N_C_** | **P (%)** | **N_C_** | **P (%)** |
| ( -∞, 1000) | 3 | 0.00 | 56 | 0.02 | 479 | 0.13 | 1698 | 0.47 |
| [ -1.5, -1 ) | 0 | 0.00 | 0 | 0.00 | 2 | 0.00 | 6 | 0.00 |
| [ -1, -0.5 ) | 0 | 0.00 | 5 | 0.00 | 15 | 0.00 | 68 | 0.02 |
| [ -0.5, 0 ) | 344 | 0.10 | 1383 | 0.39 | 3630 | 1.01 | 10219 | 2.85 |
| [ 0, 0.5 ) | 234594 | 65.47 | 279697 | 78.06 | 287782 | 80.32 | 292062 | 81.51 |
| [ 0.5, 1 ) | 60494 | 16.88 | 39338 | 10.98 | 32357 | 9.03 | 22314 | 6.23 |
| [ 1, 1.5 ) | 20318 | 5.67 | 7635 | 2.13 | 3904 | 1.09 | 1200 | 0.33 |
| [ 1.5, 2 ) | 990 | 0.28 | 590 | 0.16 | 197 | 0.05 | 34 | 0.01 |
| [ 2, 2.5 ) | 34 | 0.01 | 47 | 0.01 | 21 | 0.01 | 13 | 0.00 |
| [ 2.5, 3 ) | 6 | 0.00 | 13 | 0.00 | 5 | 0.00 | 0 | 0.00 |
| [ 3, 3.5 ) | 0 | 0.00 | 0 | 0.00 | 1 | 0.00 | 0 | 0.00 |
| [ 1000, ∞ ) | 41513 | 11.59 | 29532 | 8.24 | 29903 | 8.35 | 30682 | 8.56 |

***Table 2:*** *Histograms of values of climate velocities for minimum temperature (T_min_, the rest as in Table 1).*

| **Pcp** | **Winter** | | **Spring** | | **Summer** | | **Autumn** | |
| --- | --- | --- | --- | --- | --- | --- | --- | --- |
| **C (km/year)** | **N_C_** | **P (%)** | **N_C_** | **P (%)** | **N_C_** | **P (%)** | **N_C_** | **P (%)** |
| [ -3.5, -3 ) | 0 | 0.00 | 0 | 0.00 | 1 | 0.00 | 0 | 0.00 |
| [ -3, -2.5 ) | 1 | 0.00 | 0 | 0.00 | 6 | 0.00 | 0 | 0.00 |
| [ -2.5, -2 ) | 1 | 0.00 | 0 | 0.00 | 6 | 0.00 | 0 | 0.00 |
| [ -2, -1.5 ) | 0 | 0.00 | 1 | 0.00 | 30 | 0.01 | 0 | 0.00 |
| [ -1.5, -1 ) | 2 | 0.00 | 13 | 0.00 | 38 | 0.01 | 0 | 0.00 |
| [ -1, -0.5 ) | 5 | 0.00 | 45 | 0.01 | 166 | 0.05 | 6 | 0.00 |
| [ -0.5, 0 ) | 10003 | 2.79 | 131973 | 36.83 | 267756 | 74.73 | 5168 | 1.44 |
| [ 0, 0.5 ) | 344514 | 96.15 | 226146 | 63.12 | 89993 | 25.12 | 351942 | 98.23 |
| [ 0.5, 1 ) | 3006 | 0.84 | 108 | 0.03 | 220 | 0.06 | 884 | 0.25 |
| [ 1, 1.5 ) | 565 | 0.16 | 8 | 0.00 | 56 | 0.02 | 218 | 0.06 |
| [ 1.5, 2 ) | 182 | 0.05 | 0 | 0.00 | 20 | 0.01 | 51 | 0.01 |
| [ 2, 2.5 ) | 16 | 0.00 | 1 | 0.00 | 4 | 0.00 | 15 | 0.00 |
| [ 2.5, 3 ) | 1 | 0.00 | 0 | 0.00 | 0 | 0.00 | 3 | 0.00 |
| [ 3, 3.5 ) | 0 | 0.00 | 1 | 0.00 | 0 | 0.00 | 6 | 0.00 |
| [ 3.5, 4 ) | 0 | 0.00 | 0 | 0.00 | 0 | 0.00 | 2 | 0.00 |
| [ 4, 4.5 ) | 0 | 0.00 | 0 | 0.00 | 0 | 0.00 | 1 | 0.00 |

***Table 3:*** *Histograms of values of climate velocities for precipitation (PcP, the rest as in Table 1).*

**Programs**

MATLAB codes used for analysis in this paper (with references to external codes) can be found at <https://github.com/TeranIvy/ClimateVelocityPrograms>

1 Kosanic, A., Harrison, S., Anderson, K. & Kavcic, I. Present and historical climate variability in South West England. *Climatic Change*, 1-17, doi:10.1007/s10584-014-1101-8 (2014).

2 Río, S. d., Herrero, L., Fraile, R. & Penas, A. Spatial distribution of recent rainfall trends in Spain (1961–2006). *International Journal of Climatology* **31**, 656-667, doi:10.1002/joc.2111 (2011).

3 Wang, Q.-x., Fan, X.-h., Qin, Z.-d. & Wang, M.-b. Change trends of temperature and precipitation in the Loess Plateau Region of China, 1961–2010. *Global and Planetary Change* **92–93**, 138-147 (2012).

4 Burkey, J. *A non-parametric monotonic trend test computing Mann-Kendall Tau, Tau-b, and Sen’s Slope written in Mathworks-MATLAB implemented using matrix rotations.*, <<http://www.mathworks.com/matlabcentral/fileexchange/authors/23983> > (2006).

5 Burrows, M. T. *et al.* The Pace of Shifting Climate in Marine and Terrestrial Ecosystems. *Science* **334**, 652-655, doi:10.1126/science.1210288 (2011).

6 Burrows, M. T. *et al.* Geographical limits to species-range shifts are suggested by climate velocity. *Nature* **507**, 492-495, doi:10.1038/nature12976 (2014).

7 Loarie, S. R. *et al.* The velocity of climate change. *Nature* **462**, 1052-1055 (2009).

8 Patton, A., Politis, D. N. & White, H. Correction to “Automatic Block-Length Selection for the Dependent Bootstrap” by D. Politis and H. White. *Econometric Reviews* **28**, 372-375, doi:10.1080/07474930802459016 (2009).

9 Politis, D. N. & Romano, J. P. The Stationary Bootstrap. *Journal of the American Statistical Association* **89**, 1303-1313, doi:10.1080/01621459.1994.10476870 (1994).

10 Politis, D. N. & White, H. Automatic Block-Length Selection for the Dependent Bootstrap. *Econometric Reviews* **23**, 53-70, doi:10.1081/ETC-120028836 (2004).
